# Doctors' Wills

**Published:** 1914-04-25

**Authors:** 


### Doctors' Wills.

Fleet-Surgeon Alfred Hutton Jeremy, M.B., R.N., of Bournemouth, and of H.M.S. *Implacable*, who died intestate, has left £18,238.

Dr. John Knox, M.D., Bakewell, Derbyshire, formerly Surgeon-Lieutenant-Colonel 2nd V.B. Sherwood Foresters, senior surgeon to the Bakewell Dispensary, has left estate valued at £7,610.

Mr. James Dickson, L.R.C.P., F.R.C.S. Edin., of Mayfield House, Long Eaton, Derbyshire, surgeon, a member of the British Medical Association, and for several years vaccination officer to the Shardlow Board of Guardians, has left £3,260.

